# Inter-polysomal coupling of termination and initiation during translation in eukaryotic cell-free system

**DOI:** 10.1038/srep24518

**Published:** 2016-04-14

**Authors:** Evgeny A. Sogorin, Sultan Ch. Agalarov, Alexander S. Spirin

**Affiliations:** 1 Institute of Protein Research, Russian Academy of Sciences, Pushchino, Moscow region, Russia

## Abstract

The recording of the luciferase-generated luminescence in the eukaryotic cell-free translation system programmed with mRNA encoding firefly luciferase (Luc-mRNA) showed that the addition of free exogenous mRNAs into the translation reactor induces the immediate release of the functionally active protein from the polyribosomes of the translation system. The phenomenon did not depend on the coding specificity of the added free mRNA. At the same time it depended on the “initiation potential” of the added mRNA (including the features that ensure the successful initiation of translation, such as the presence of the cap structure and the sufficient concentration of the added mRNA in the translation mixture). The phenomenon also strictly depended on the presence of the stop codon in the translated mRNA. As the above-mentioned features of the added mRNA imply its activity in initiation of a new translation, the experimental data are found in agreement with the scenario where the molecules of the added mRNA interact by their 5′-ends with terminating and recycling ribosomes, stimulating the release of the complete polypeptides and providing for the initiation of a new translation.

The translation initiation phase starts from the formation of the 43S pre-initiation complex comprising the 40S ribosomal subunit in associating with a number of proteins (called initiation factors) and Met-tRNAi (reviewed in ref. 1). This complex binds with mRNA, usually with its capped 5′-terminal region (called also 5′-untranslated region or 5′-UTR). Typically in the case of eukaryotic mRNAs the 5′-terminal cap structure serves for the binding of the initiation ribosomal particle to mRNA. The mRNA-bound 43S initiation complex slides along the mRNA chain, usually demonstrating the energy-dependant unidirectional motion in the 5′ to 3′ direction along the 5′-untranslated region (5′-UTR) of mRNA. The moving initiation 43S ribosomal complex scans the nucleotide sequence of the 5′-UTR until it recognizes the start codon^2^,^3^,^4^. In some cases the energy-independent scanning of the UTR can be observed^5^.

The recognition of the start codon during the sliding of the ribosomal 43S complex along the untranslated region put an end to the sliding and induces a fundamental transformation into the ribosomal 48S complex. Further the following steps are fulfilled: (1) the re-association of the small ribosomal subunit with the large ribosomal subunit into the full 80S ribosome; (2) the setting of the initiator aminoacyl-tRNA (Met-tRNAi) into the P-site of the ribosome; (3) the adjusting of the vacant A-site for codon-dependent binding of the next aminoacyl-tRNA that begins the elongation phase of the translation process. The codon-by-codon movement of the ribosome along the coding region of mRNA in the 5′ to 3′ direction is coupled with the amino acid additions to the polypeptide synthesized by the ribosome, resulting in the polypeptide elongation.

Termination starts when a moving translating ribosome, after reading all the coding sequence of the mRNA, reaches and recognizes the stop codon^6^,^7^,^8^. The termination process in a eukaryotic ribosome involves two termination factors: the stop-codon-binding protein eRF1 and the GTP-dependent protein eRF3^9^, but strictly sequentially, ribosome by ribosome, in accordance with their order along the mRNA chain. After the recognition of the stop codon at the end of the coding sequence the ribosome-bound peptidyl-tRNA is hydrolyzed into tRNA and polypeptide, resulting in the release of the full-length (complete) polypeptide from the terminating ribosome. As shown for a number of globular proteins, their polypeptide chains are folding into functionally active globules during translation (the so-called cotranslational folding); the firefly luciferase was among the first examples of such a case^10^.

In the present work, in order to retrace the process of translation, and especially the immediate post-termination events, we used the methodology of the cell-free synthesis of the firefly luciferase, which allows measuring its enzymatic activity directly in the reaction mixture^10^. It has been found that the release of the full-length active protein from translating polyribosomes depends on the presence of free mRNAs in the reaction milieu: the addition of free mRNAs to the translation system during its high synthetic activity phase has been shown to induce the immediate release of a portion of the self-folded globules of the active protein (luciferase) from translating polyribosomes.

## Results

Firefly luciferase is the enzyme that catalyzes ATP-dependent conversion of the luciferin substrate into its oxidized form; the conversion is accompanied by the emission of light. Thus, the activity of the protein-synthesizing system and the activity changes in the course of the protein (luciferase) synthesis were continuously recorded during incubation of the translation mixture directly in the luminometer cell. The following results have been obtained.

**Figure 1:**
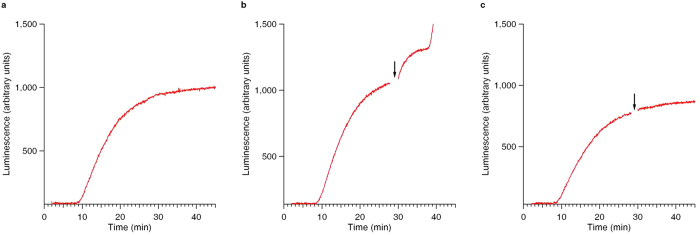
Time course of the synthesis and accumulation of functionally active luciferase in the cell-free translation reactor. The translation was started and programmed by the addition of Luc-mRNA up to its final concentration of 50 pmol per ml. (**a**) The time course curve of the luciferase synthesis. Three stages can be noted: (1) the initial lag with duration of about 8–9 min (this time corresponds to the transit time of the moving ribosome along the mRNA), when no complete active proteins appeared yet; (2) the productive period of the active protein synthesis during *ca*. 20 min; and (3) the period of the synthesis deceleration passing into plateau. (**b**) Time course of the accumulation of functionally active luciferase in the translation reactor. The translation system was initially started by the addition of Luc-mRNA up to its final concentration of 50 pmol per ml. The capped free Luc-mRNA was added into the active translation system (“post-start mRNA addition”) up to the final concentration of 50 pmol per ml during the productive protein synthesis stage, just before the plateau stage, as marked by vertical arrow. (**c**) All was the same as in Fig. 1b, but total transfer RNA was added as a control to the translating system instead of mRNA.

The addition of free mRNA to the cell-free translation system (at the time during the high productivity phase) induced the immediate release of complete active protein from the translating polyribosomes of the system. Figure 1 shows the time course curves of the synthesis of the firefly luciferase in the cell-free translation system using the luciferase-encoding mRNA (called Luc-mRNA here and further) for translation. The time between the start of the translation and the appearance of the first active product was about 8 min (Fig. 1a–c), this corresponding to the transit time for the synthesis of the firefly luciferase under the translation conditions used. As seen in Fig. 1a, the time course curve of the luminescent protein accumulation reached the plateau by about the 30^th^ minute of incubation. Figure 1b demonstrates the unexpected phenomenon: when free mRNA was added into the cell-free translation reactor during active translation process, two sequential jumps of the luminescence intensity were recorded. The first one was a short-time rise of the protein synthesis, occurred immediately upon the post-start addition of the free mRNA, without any time lag (Fig. 1b). It should be recalled that the newly synthesized firefly luciferase can display its activity only upon leaving the ribosome^10^. This implies that the luciferase released during the first rise (between the 30^th^ and 40^th^ minutes of the translation run, see Fig. 1b) had already passed through the termination phase, and its polypeptide chains were already complete by the time of the post-start mRNA addition. Hence, the first rise reflects the release of a significant portion of the luciferase activity, that means the discharge of the respective portion of the complete luciferase polypeptides (self-folded into the active protein globules) from polysomal ribosomes, occurred for a substantially less time (within one minute) than the time required for the ribosomal synthesis of the full-size luciferase (the latter should be equal to the transit time that was about 8 min in this case). Hence, it should be the pre-synthesized post-termination luciferase that was released from the polyribosomes upon the addition of the free mRNA (Fig. 1b). It is important to notice that the duration of the jump of the luciferase activity was short, just about 2 min (between the 31^st^ and the 33^rd^ min, Fig. 1b). Then, in about 6–8 min after the jump start a new abrupt rise of the luciferase activity in the translation reactor occurred (started at the 38^th^ min, Fig. 1b). The interval between the two rises was equivalent to the transit time for the synthesis of the luciferase polypeptides by translating ribosomes in our cell-free translation reactor, this implying that the second rise reflected the beginning of a new translation run, involving the added free mRNAs as templates for translation. Thus, the added free mRNA caused two events in the translation system used. The first was the induction of the instant release of the already complete full-length and obviously self-folded polypeptides from the translating polysomal ribosomes, thus demonstrating their successful passing of both the elongation and the termination phases of the translation process. The second event should be the initiation when the syntheses of new polypeptide chains became resumed on the added free mRNAs.

To exclude the possibility that the induced changes of luciferase luminescence could be a non-specific reaction to the presence of any RNA as a chemical substance, a control experiment was performed. Figure 1c shows that when the total tRNA was added instead of mRNA, no effects of the luminescence intensity changes during incubation of the translation mixture were detected.

The effect of the induction of luciferase activity rise by the post-start addition of free mRNA to the translation mixture could be also observed when the addition was made at the earlier stages of the Luc-mRNA translation. Supplementary Fig. 1 demonstrates the result of the experiment when the post-start addition of free mRNA to the cell-free translation reactor was done at the 12^th^–14^th^ min of translation, i.e. at the time during the initial linear course of the luciferase accumulation, about 15 min before the plateau. Although the magnitudes and the proportions of the induced rises of the luciferase activity in this case significantly differed from those described in Fig. 1b, the principal similarity of the effects is evident.

**Figure 2:**
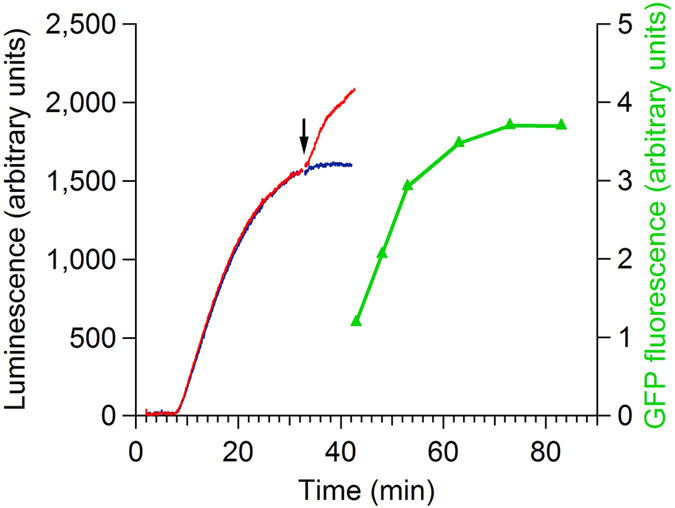
Time courses of the synthesis of functionally active luciferase (left) and Green Fluorescent Protein (GFP, right) in the wheat germ cell-free translation system. The translation was started and programmed by Luc-mRNA (the final concentration of 50 pmol/ml). The GFP mRNA was added into the active translation system just before the plateau stage as marked by vertical arrow up to the final concentration of 400 pmol/ml (red curve). No mRNA was added in the case of the parallel control translation reaction (blue curve). Note that in response to the addition of the GFP mRNA the rise of luciferase luminescence was observed (see red curve after the arrow). In 10 min after the GFP mRNA addition, aliquots were taken to measure fluorescence of the synthesized GFP (green curve).

The effect of the short-time rise induction of the luciferase synthesis by the post-start addition of free mRNA to the translation mixture did not depend on the coding specificity of the added free mRNA. Figure 2 shows that the induction of the luciferase synthesis rise during translation of Luc-mRNA could be caused by the addition of a translatable mRNA of other coding specificity; in this case the free mRNA encoding the Green Fluorescence Protein (GFP), served as an inducer of the luciferase activity rise (compare Fig. 2 with Fig. 1b).

**Figure 3:**
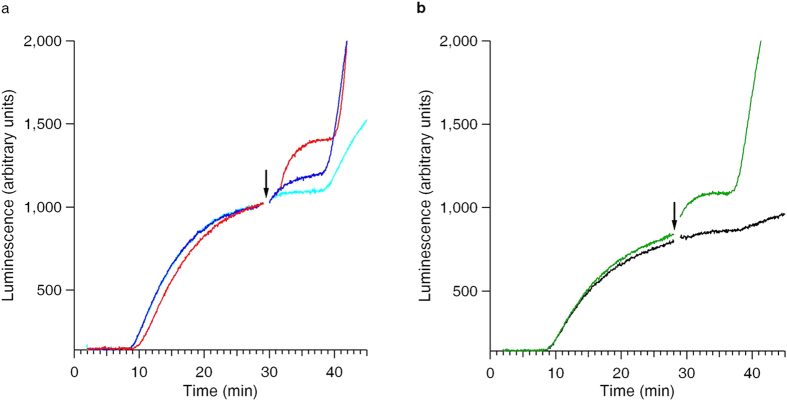
Dependence of the protein release stimulation effect on the added free mRNA concentration and on the presence of the cap structure. (**a**,**b**) The translation system was started and run as described in Fig. 1 legend. (**a**) The free uncapped Luc-mRNA with omega leader sequence (see Methods section) was added to the translation mixture at the time before the plateau, the added mRNA final concentrations being adjusted up to 50 pmol/ml (cyan curve), 100 pmol/ml (blue curve) and 250 pmol/ml (red curve) in three parallel translation runs, respectively. (**b**) The capped (green curve) and uncapped (black curve) Luc-mRNAs with β-globin leader sequence were added to the translation system up to their final concentration of 50 pmol/ml, during the active protein (luciferase) synthesis stage, at the time point before the plateau stage, as described in Fig. 1b legend. The time point of the post-start additions of the free mRNA is marked by vertical arrows.

The effect of the induction of the luciferase activity rise by the post-start addition of free mRNA to the translation mixture correlates with the initiation potential of the added mRNA. Supplementary Figs 2a and 3a illustrate how the concentrations of the added free mRNA in translation mixtures (the Luc-mRNA in this case) influence on the translation productivity of the protein synthesis systems. It is likely that the initiation stage of translation is the most responsible for the translation potential of mRNAs. Supplementary Fig. 2b evidences of the decisive role of the initiation of translation in more direct way: it demonstrates that the cap structure, immediately participating in initiation, provides for good translation, whereas the absence of the cap structure retards the initiation, sometimes to the insignificant level. Figure 3a and Supplementary Fig. 3a–c show that the induction of the luciferase activity rises, or jumps, required a sufficient concentration of the added free mRNA, and the higher was their final concentration in the translation mixture, the more effective was the induction.

Figure 3b clearly displays how the absence of cap structure at the 5′-end of the post-start added mRNA fully deprives it of the capability to induce the luciferase activity jumps.

**Figure 4:**
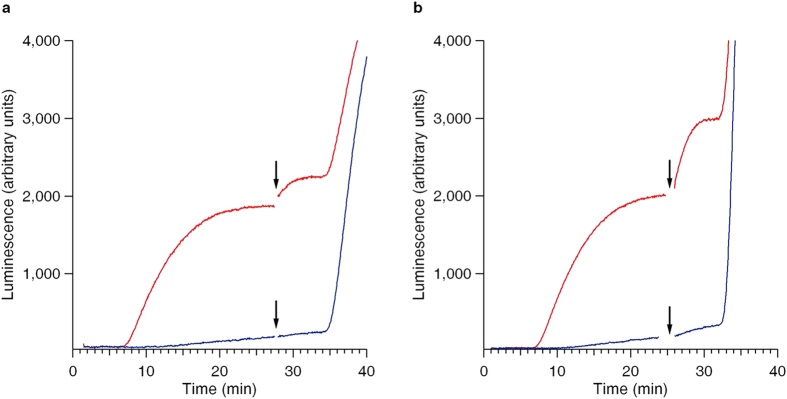
Dependence of the protein release stimulation effect on the presence of the stop codon in the primary template mRNA of the cell-free system. Note that the release of the active protein upon the post-start addition of free mRNA was found to be insignificant in the case of the mRNA deprived of the stop codon. (**a**,**b**) The translation system was started by the addition of the Luc-mRNA up to its final concentration of 50 pmol/ml (red curves), and the same mRNA in the same concentration, but deprived of the stop codon (blue curves). The free Luc-mRNA was added to the translation mixture up to its final concentrations of 100 pmol/ml (**a**) and 250 pmol/ml (**b**). The time points of the post-start mRNA additions are marked by vertical arrows.

The induction of the rise of luciferase activity by the post-start addition of free mRNA fully depends on the presence of stop codon in the translated mRNA of the translation system. In the process of translation it is the recognition of stop codon by a translating ribosome that ceases the elongation phase and starts the termination process. Hence, as far as in our experiments the addition of free exogenous mRNAs to translation mixture induced termination, we were challenged to perform a series of control experiments with the mRNAs devoid of stop codons. Supplementary Fig. 4 shows that the translation of Luc-mRNA devoid of a stop codon is of low productivity as compared with that of normal mRNAs. Nevertheless, as seen in the Supplementary Fig. 5, the mRNAs without a stop codon can successfully form polyribosomes in translation systems. Figure 4 is among the most important illustrations of the absolute requirement of the stop codon for the stimulation of the translation termination by the added free mRNAs.

## Discussion

The intriguing questions that arise from the fact of the mRNA-induced release of complete, functionally active protein from the translated polyribosomes concern the localization and the form of existence of this protein within polyribosomes before the induced release. It cannot be excluded that a fraction of the terminating ribosomes in the translating polyribosomes may retain the non-hydrolyzed peptidyl-tRNAs, and the contact with the added free mRNA provokes the hydrolysis thus leading to the release of the active protein. An interesting hypothetical alternative might be the temporary retention of the released protein outside the terminated ribosomes, but still inside the 3D-structured polyribosomes of the helical type^11^. In any case, the fact of the necessity of stop codon for inducing the polypeptide release by the addition of exogenous mRNA to the translating polyribosomes evidences that the release phenomenon reflects the conventional termination process.

Recently, using ribosome profiling it was shown^12^ that there is a tendency of accumulation of translating ribosomes in the vicinity of the stop codon relative to the rest coding part of the mRNA. When the ATP-bound cassette ABCE1/Rli1 level was diminished, the peak of the ribosome footprint at stop codons was greatly magnified, and another (smaller) peak appeared *ca*. 30 nt upstream. The authors explained these phenomena by the termination/recycling delay at the stop codon, which accompanied by queuing of the trailing elongating ribosomes behind those stalled at the stop codon^12^. It is not excluded that the situation is similar in our experiments, where a jam-up may occur in the region of the stop codon. It is known that Rli1/ABCE1 associates with initiation factors eIF2, eIF3, eIF5 and promotes the pre-initiation 43S complex assembly^13^,^14^. It was supposed that ABCE1 could be a part of the 43S complex via interaction with eIF3^13^. In our initial experimental conditions, where mRNA was highly deficient relative to ribosomes (molar ratio is 1: 20–30), it is likely that most of ABCE1 is bound within the 43S complex. This may explain the hindrance to its participation in the processes of termination or recycling of the ribosomes. The addition of free exogenous mRNA induces the involvement of the pre-initiation 43S complexes in the translation initiation process and thus leads to the release of ABCE1. Afterwards, ABCE1 promotes termination or recycling of the stalled ribosomes.

## Methods

### The mRNA preparations

In this study, for all our experiments, we used the recombinant mRNA, called here Luc mRNA, consisting of the translated region encoding the firefly luciferase and the so-called omega sequence from tobacco mosaic virus RNA as the 5′-proximal untranslated region, i.e. the leader sequence of a recombinant construct. In the recombinant constructs the leader omega sequence has been shown to enhance translation of mRNAs^15^ and to support translation of the mRNAs without cap structure^16^.

All procedures for the isolation and purification of mRNAs were described in detail elsewhere^17^. Capping of the mRNAs was performed using the Vaccinia Capping System (NEB), with the following purification in MicroSpin™ G-50 columns (GE Healthcare). The procedures of preparations of the mRNA constructs and the plasmids used were described in the previous publications as referred to in the following list: the Luc-mRNA containing omega-leader^18^, the mRNA carrying the obelin leader and the GFP encoding region^19^, the mRNA with β-globin mRNA leader^20^. The Luc-mRNA devoid of stop codon was prepared by substitution of UCA triplet for UAA *ochre* codon; the construct was kindly provided by Dr. V.A. Shirokov.

### *In situ* monitoring of luciferase synthesis

The method was described in detail previously^18^. Translation was performed in the cell-free translation system based on the wheat germ extract using the batch format according to the published protocol^21^. The translation system was started by addition of the mRNA to be translated; the reaction mixture was incubated at 25 °C in the temperature-controlled cell of the luminometer (“Hemilum-12”, Institute of Cell Biophysics, RAS). The intensity of light emission was measured continuously (every 2.5 s) by collecting the streaming data with the computer as a kinetic curve. In the case of the post-start addition of mRNA, the luminometer was temporarily turned off, the mRNA was added into the cell, and the mixture was stirred. Then, as soon as the samples were returned into the luminometer, it was switched on again and the collection of luminescence data was continued.

## Additional Information

**How to cite this article**: Sogorin, E. A. *et al*. Inter-polysomal coupling of termination and initiation during translation in eukaryotic cell-free system. *Sci. Rep*. **6**, 24518; doi: 10.1038/srep24518 (2016).

## Supplementary Material

Supplementary Information
