# Supplementary material for: Inter-polysomal coupling of termination and initiation during translation in eukaryotic cell-free system
Source: Sci Rep. 2016 Apr 14;6:24518. doi: 10.1038/srep24518 (PMC4830951; doi:10.1038/srep24518)
Supplement: Supplementary Information [file srep24518-s1.doc]

**Supplementary information**

**Inter-polysomal coupling of termination and initiation
during translation in eukaryotic cell-free system**

Evgeny A. Sogorin, Sultan Ch. Agalarov and Alexander S. Spirin

*Institute of Protein Research, Russian Academy of Sciences,
Pushchino, Moscow region, Russia*


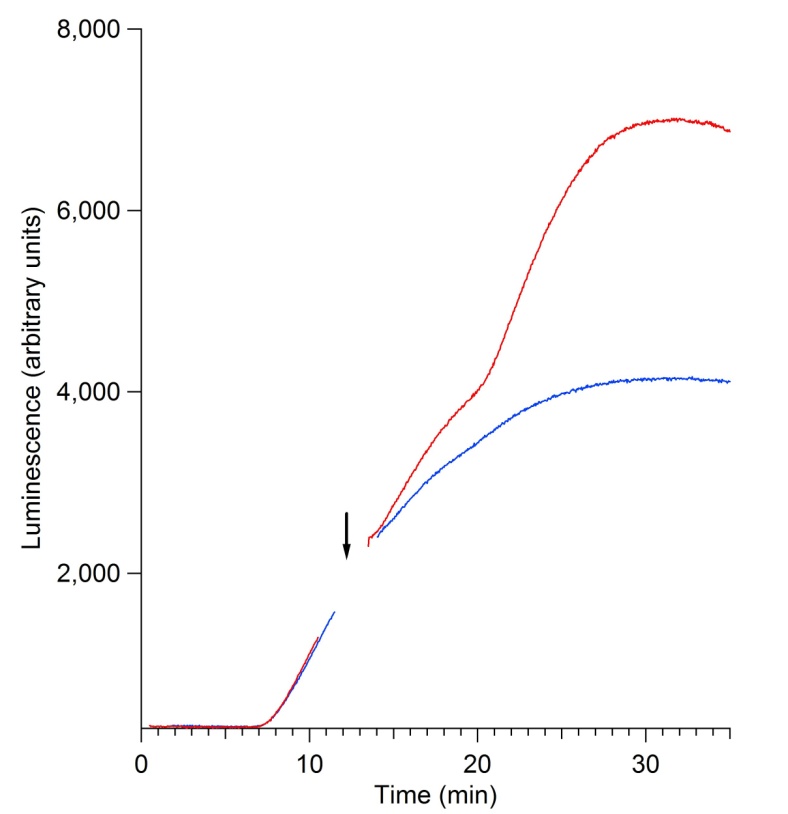


**Supplementary Figure 1. Time course of the synthesis of functionally active luciferase in the cell-free translation system.** The translation system was started by the addition of Luc-mRNA up to its final concentration of 125 pmol/ml. The post-start portion of free Luc-mRNA was added into the active translation system up to the final concentration of 125 pmol/ml during the initial linear course of the luciferase accumulation, as marked by vertical arrow (red curve). No post-start mRNA was added in the case of the parallel control translation reaction (blue curve).


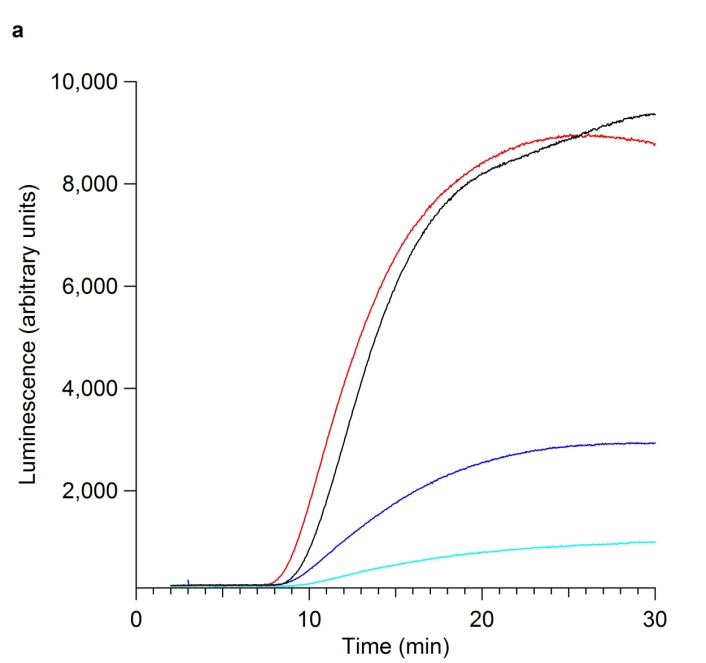

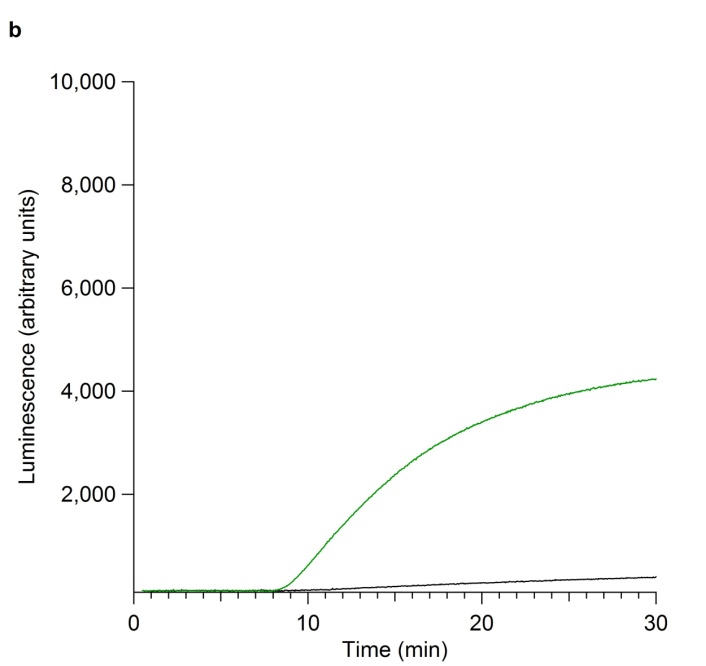


**Supplementary Figure 2. Time course of the synthesis of functionally active luciferase** **in the cell-free translation system.** (**a**) Luc-mRNA containing omega leader at different final concentrations: 50 pmol/ml (cyan), 100 pmol/ml (blue), 250 pmol/ml (red), and 500 pmol/ml (black). (**b**) Luc-mRNA containing the β-globin leader (50 pmol/ml): uncapped (black), capped (green).


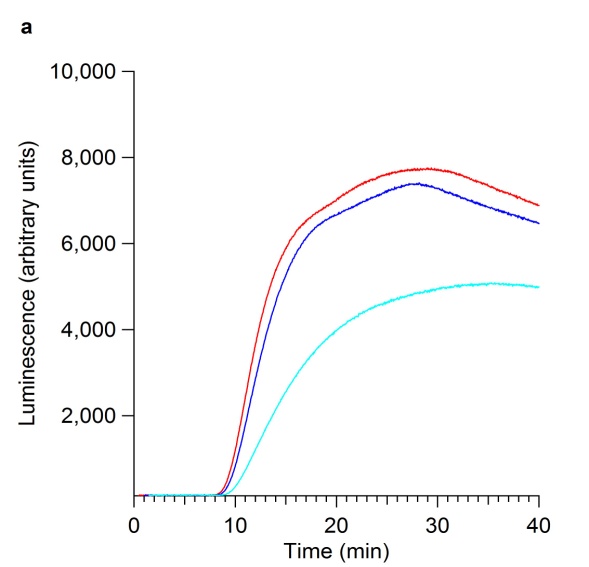

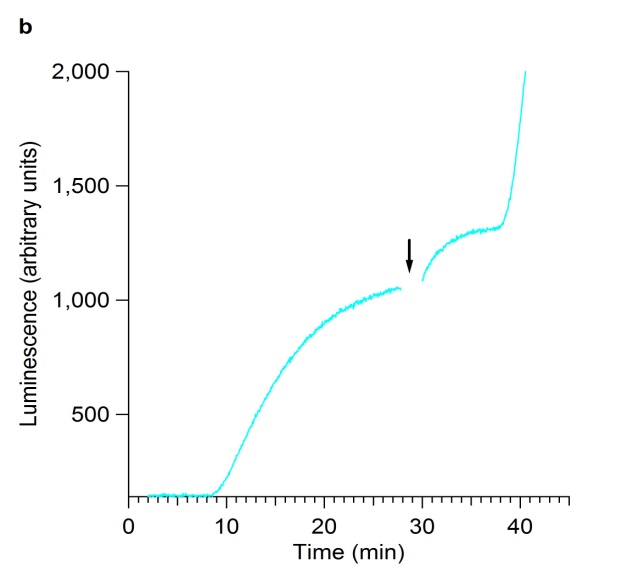

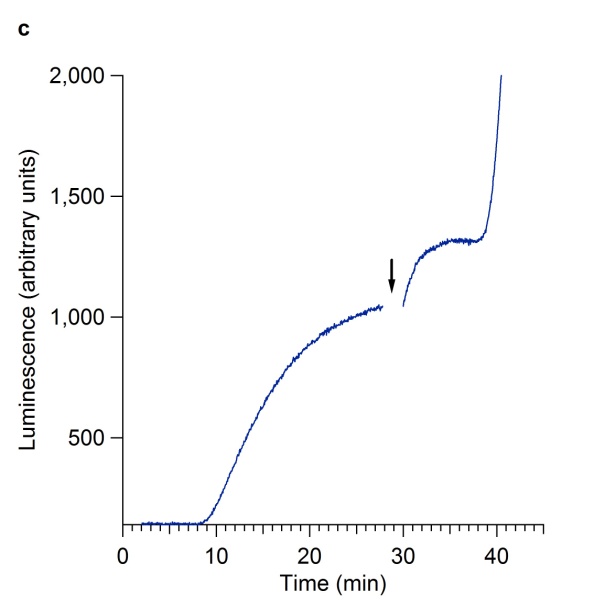

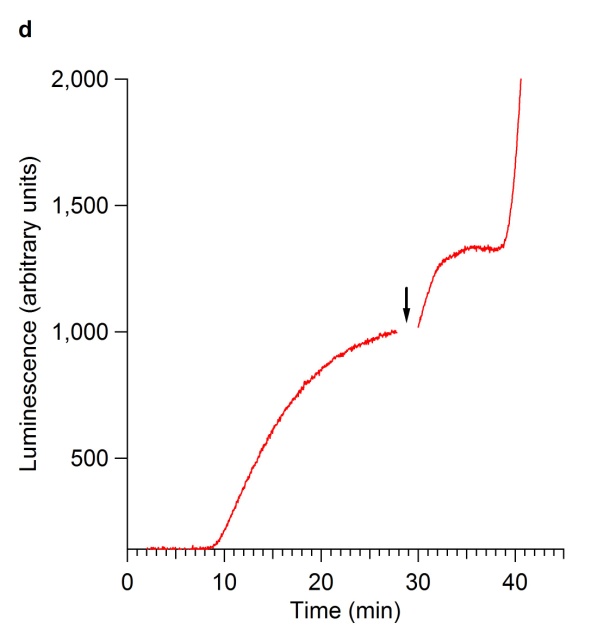


**Supplementary Figure 3. Dependence of the protein release stimulation effect on the added free mRNA concentration.** (**a**) Time courses of the synthesis of functionally active luciferase; Luc-mRNA containing capped omega leader at different final concentrations: 50 pmol/ml (cyan curve), 100 pmol/ml (blue curve), and 200 pmol/ml (red curve). (**b-d**) The translation system was started by the addition of Luc-mRNA up to its final concentration of 50 pmol/ml. The post-start portion of free Luc-mRNAs with capped omega leader were added into the active translation system just before the plateau stage, as marked by vertical arrows, up to their final concentrations (increasing the “initiation potential” of the added Luc-mRNA): (**b**) 50 pmol/ml, (**c**) 100 pmol/ml, (**d**) 200 pmol/ml.


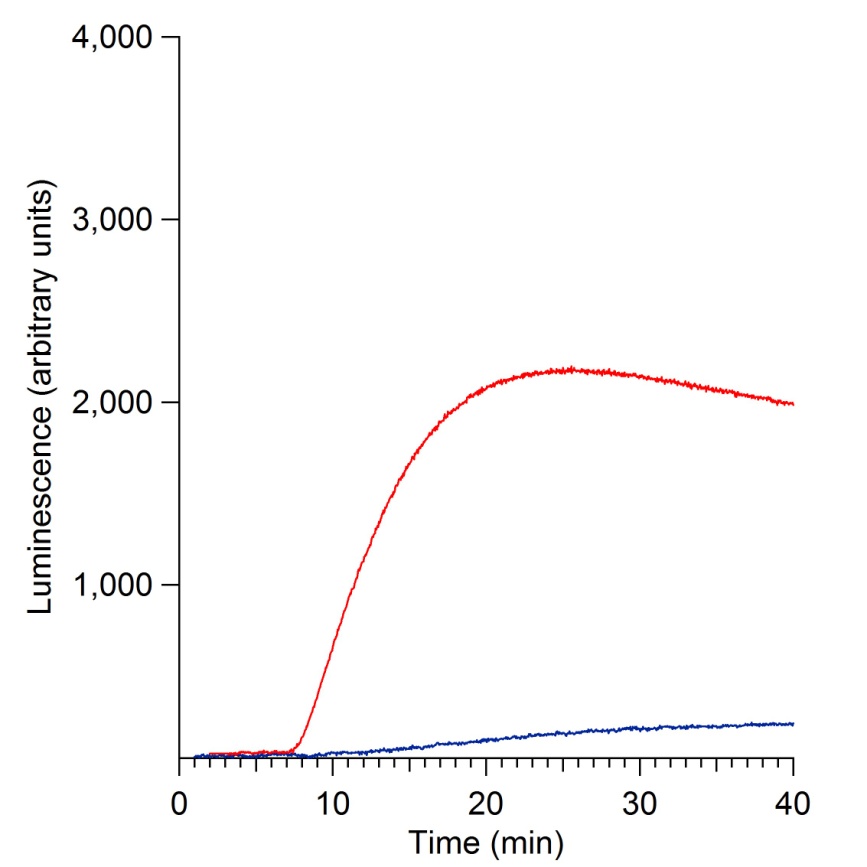


**Supplementary Figure 4. Dependence of the synthesis of functionally active luciferase on the presence of the stop codon in the translated mRNA.** Translation of the Luc-mRNA, 50 pmol/ml (red curve), and the same Luc-mRNA deprived of the stop codon, 50 pmol/ml (blue curve).


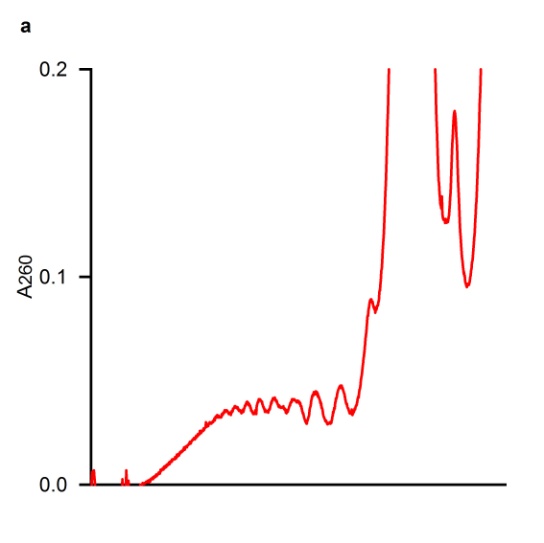

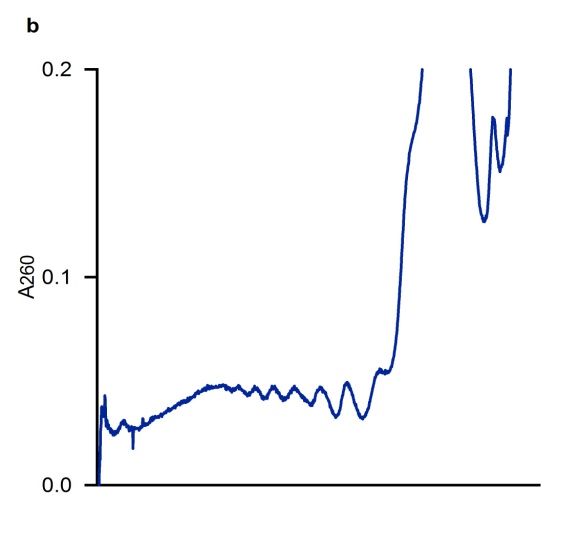


Sedimentation

**Supplementary Figure 5. Sucrose gradient sedimentation analysis of the translation mixture after 20 min incubation under conditions of active protein synthesis.** The synthesis of the firefly luciferase was performed in the cell-free translation system programmed with the Luc-mRNA (see the **Methods** section) during 20 min; the reaction was terminated by addition of cycloheximide, and the sample was centrifuged for 2 h in SW-41 rotor at 37,000 rpm, +4°C. (**a**) The sedimentation profile of the polyribosomes formed during 20 min translation of the original Luc-mRNA. (**b**) The sedimentation profile of the polyribosomes formed during 20 min translation of the Luc-mRNA deprived of the stop codon. Note that the fast-sedimentating UV-absorbing material seen at the centrifuge tube bottom is caused by the overloaded polyribosomes owing to the impossibility of the normal termination.

**Supplementary text: Discussion material**

In our studies of interactions between eukaryotic polyribosomes during translation in cell-free systems the addition of free exogenous mRNA into the translation reactor revealed an unexpected phenomenon: the immediate release of the full-size, functionally active protein from the translating polyribosomes was recorded. Recently we have shown the partial disassembly of the pre-existing (old) polyribosomes occurring simultaneously with the formation of new polyribosomes on the free mRNA added into the translation system (ref. S1). Accordingly, both phenomena may be explained in terms of the same mechanism. We suggest two possible mechanisms: direct and indirect action of the added mRNA.

**Direct action of the added mRNA: three different cases**

On the whole, this study sets thinking about different cases of the translation re-initiation in functional behavior of eukaryotic polyribosomes. There are three cases that can be considered on the subject of possible similarities and differences between the mechanisms of the realization of the translation reinitiation function. Case 1 is the classical re-initiation process during sequential translation of ORFs in bicistronic or polycistronic polyribonucleotide messages (ref. S2-5). Case 2 is the case of eukaryotic circular polyribosomes, where the 5' and 3' terminal regions of the same mRNA are joint together in the way that allows ribosomes to move along the circularized mRNA, thus resulting in the so-called “circular translation” of the mRNA (ref. S6-7). Case 3 could be the new initiation when the stop-codon-induced termination at one monocistronic mRNA is found to be coupled with the initiation at another mRNA in vicinity.

In the first two cases the reinitiation is an *intra-*polynucleotide event, whereas the case 3 is an *inter*-polynucleotide action: the addition of the free mRNA into the translation system leads to contact and plausible coupling of the 5'-terminal initiation region of this new mRNA with the terminating region of the translated original (“old”) polysomal mRNA. In other words, the initiation 5'-end of the added free mRNA is supposed to attack and draw on itself the stop-codon-neighboring 3'-proximal region of the translating polysomal mRNA resulting in initiation of translation of the added mRNA by the ribosomes from the translating polysomal mRNA. It cannot be excluded that in this case the circular conformation of polyribosomes can be supported by the intra-polyribonucleotide coupling, instead of the cap-poly(A) bridge.

**Indirect action of the added mRNA:**

**pre-initiation 43S complexes in the translation initiation process**

The second possible mechanism predicts indirect action of the newly added mRNA. Recently, using ribosome profiling it was shown that there is a tendency of accumulation of translating ribosomes in the vicinity of the stop codon relative to the rest coding part of the mRNA (ref. S8). When the Rli1/ABCE1 level was diminished, the peak of the ribosome footprint at stop codons was greatly magnified, and another (smaller) peak appeared *ca*. 30 nt upstream. The authors explained these phenomena by the termination/recycling delay at the stop codon, which accompanied by queuing of the trailing elongating ribosomes behind those stalled at the stop codon. It is not excluded that the situation is similar in our experiments, where a jam-up may occur in the region of the stop codon, thus preventing termination or recycling of the ribosomes. In this case, the question arises how the ABCE1 deficiency could be explained. It is known that Rli1/ABCE1 associates with initiation factors eIF2, eIF3, eIF5 and promotes the pre-initiation 43S complex assembly (ref. S9-10). It is supposed that ABCE1 can be a part of the 43S complex via interaction with eIF3 (ref. S9). In our initial experimental conditions the pre-initiation 43S complexes in the translation initiation process, where mRNA was highly deficient relative to ribosomes (molar ratio is 1: 20-30), it is likely that most of ABCE1 is bound within the 43S complex. This may explain the hindrance to its participation in the processes of termination or recycling of the ribosomes.

Thus, the addition of free exogenous mRNA induces the involvement of the pre-initiation 43S complexes in the translation initiation process and leads to the release of the ATP-bound cassette ABCE1. Afterwards, ABCE1 promotes termination or recycling of the stalled ribosomes.

**References**

S1. Sogorin, E.A., Agalarov, S.Ch. & Spirin, A.S. Formation of new polysomes on free mRNAs in a cell-free translation system is accompanied by partial disassembly of previously formed polysomes. *Biochemistry (Moscow)* **80,** 1327-1330 (2015)*.*

S2. Jackson, R.J., Hellen, C. U. T. & Pestova, T. V. Termination and post-termination events in eukaryotic translation. *Adv. Protein Chem. Struct. Biol*. **86**, 45-93 (2012).

S3. Dever, T. E., & Green, R. The elongation, termination, and recycling phases of translation in eukaryotes. *Cold Spring Harb. Perspect. Biol.* **4**, a013706 (2012).

S4. Skabkin, M. A., Skabkina, O. V., Hellen, C. U. T. & Pestova, T. V. Reinitiation and other unconventional posttermination events during eukaryotic translation.  *Molecular Cell* **51**, 1–16 (2013).

S5. Kozak, M. Effects of intercistronic length on the efficiency of reinitiation by eucaryotic ribosomes. *Mol. Cell. Biol.* **7**, 3438–3445 (1987).

S6. Philipps, G. R. Haemoglobin synthesis and polysomes in intact reticulocytes.  *Nature*, **205**, 567-570 (1965).

S7. Afonina, Z. A., Myasnikov, A. G., Shirokov, V. A., Klaholz, B. P., & Spirin, A. S. Conformation transitions of eukaryotic polyribosomes during multi-round translation. *Nucleic Acids Res.* **43**, 618-628 (2015).

S8. Young, D. J., Guydosh, N. R., Zhang, F., Hinnebusch, A. G., & Green, R. Rli1/ABCE1 recycles terminating ribosomes and controls translation reinitiation in 3′ UTRs In Vivo. *Cell*, **162**, 872-884 (2015).

S9. Dong, J. et al. The essential ATP-binding cassette protein RLI1 functions in translation by promoting preinitiation complex assembly. *J. Biol. Chem.* **279**, 42157-42168 (2004).

S10. Chen, Z. Q. et al. The essential vertebrate ABCE1 protein interacts with eukaryotic initiation factors. *J. Biol. Chem.* **281**, 7452-7457 (2006)
